# Cortical polarity ensures its own asymmetric inheritance in the stomatal lineage to pattern the leaf surface

**DOI:** 10.1126/science.add6162

**Published:** 2023-07-06

**Authors:** Andrew Muroyama, Yan Gong, Kensington S. Hartman, Dominique Bergmann

**Affiliations:** 1. Department of Biology, Stanford University, Stanford, CA 94305, USA; 2. Division of Biological Sciences, Department of Cell and Developmental Biology, University of California San Diego, La Jolla, CA 92093, USA; 3. Current Address: Department of Organismic & Evolutionary Biology, Harvard University, Cambridge, MA 02138, USA; 4. Howard Hughes Medical Institute, Stanford University, Stanford, CA 94305, USA

## Abstract

Asymmetric cell divisions specify differential cell fates across kingdoms. In metazoans, preferential inheritance of fate determinants into one daughter cell frequently depends on polarity-cytoskeleton interactions. Despite the prevalence of asymmetric divisions throughout plant development, evidence for analogous mechanisms that segregate fate determinants remain elusive. Here, we describe a mechanism in the *Arabidopsis* leaf epidermis that ensures unequal inheritance of a fate-enforcing polarity domain. By defining a cortical region depleted of stable microtubules, the polarity domain limits possible division orientations. Accordingly, uncoupling the polarity domain from microtubule organization during mitosis leads to aberrant division planes and accompanying cell identity defects. Our data highlight how a common biological module, coupling polarity to fate segregation via the cytoskeleton, can be reconfigured to accommodate unique features of plant development.

## Stomatal lineage cells break common division orientation rules

Asymmetric divisions establish differential cell identities by positioning daughter cells relative to fate-enforcing extrinsic signals or by segregating intrinsic fate determinants (1, 2). The development of many plant organs depends on asymmetric divisions that place daughter cells in proximity to neighbor-derived signals, such as mobile transcription factors (3, 4), hormones (5-7), or small peptides (8, 9). However, whether plants have mechanisms that ensure preferential inheritance of fate regulators remains unresolved.

**Figure 1.**
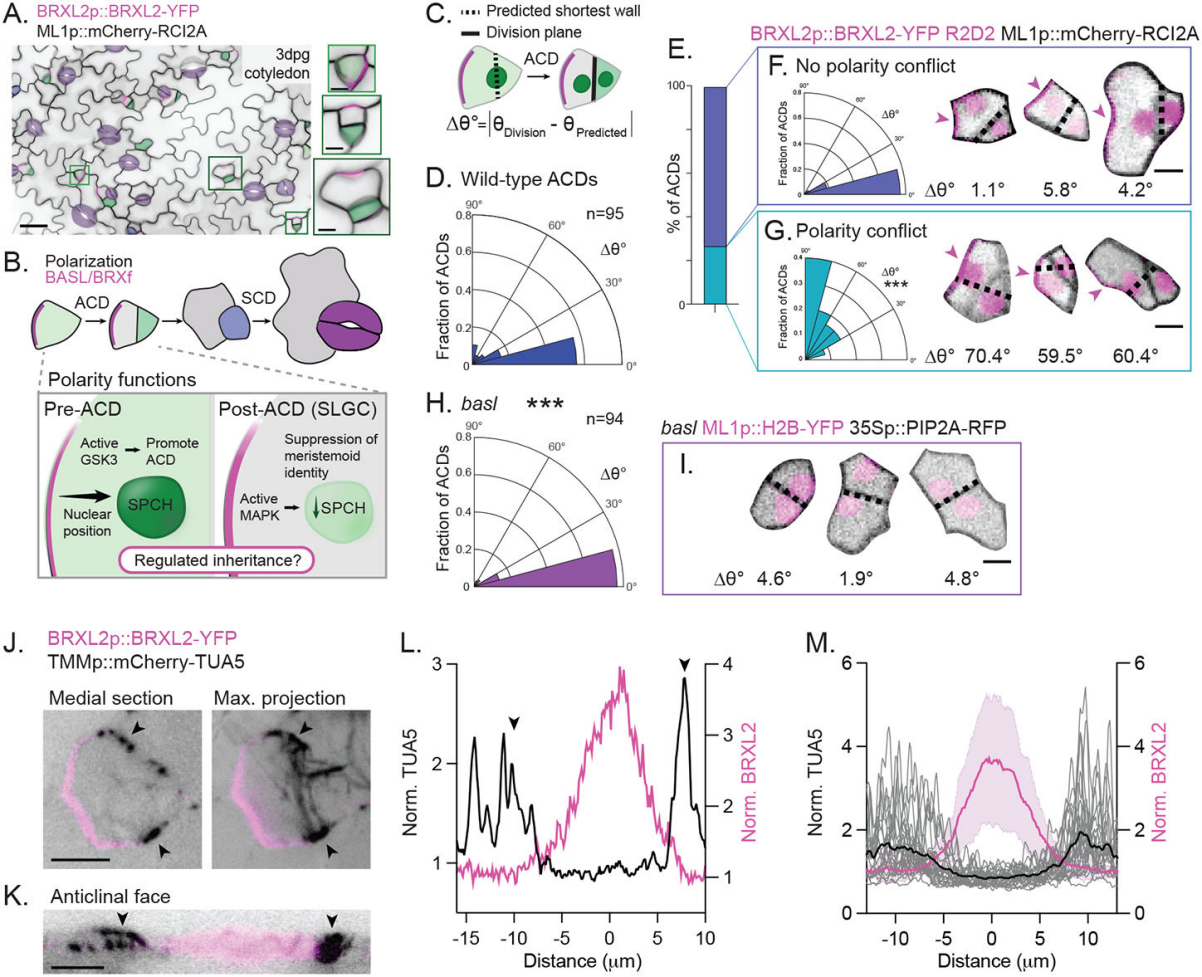
The *Arabidopsis* stomatal lineage polarity domain overrides default division rules during asymmetric cell division. A. (Left) Image of the abaxial epidermis from a 3dpg BRXL2p::BRXL2-YFP (magenta) ML1p::mCherry-RCI2A (black) cotyledon. Cells have been pseudo-colored according to the color-scheme below. Scale bar-25μm. (Right) Selected polarized cells highlighting the morphological diversity of cells in this lineage. Scale bar-5μm. B. Stomata (purple) are created through coupled divisions (asymmetric, ACD and symmetric, SCD) and fate transitions. (Top) Illustration of the major fate transitions within the *Arabidopsis* stomatal lineage; meristemoids in green, and guard mother cell in blue. (Bottom) Illustration of the known functions for the BASL/BRXf domain. Before division (in SPCH+ cells), cortical BASL/BRXf promotes ACD and instructs nuclear migration. Post-division (in stomatal lineage ground cells, SLGCs), inherited BASL/BRXf/YODA phosphorylates MAPK, leading to suppression of SPCH. Mechanisms controlling singular polarity domain inheritance are needed. C. The assay used to quantify Δθ°, the angle between the calculated shortest wall and the division plane. This assay was used for the data shown in Fig.1, D to I. For additional details, see Materials and Methods. D. Quantification of Δθ° during wild-type ACDs. n = 95 cells. E. The percent of total wild-type ACDs where polarity did (27%) or did not (73%) conflict with the predicted shortest wall. F-G. (Left) Distribution of Δθ° in ACDs where the predicted shortest wall did (G) or did not (F) conflict with the polarity domain. (Right) Three examples of ACDs from their respective classes with associated Δθ°s. The black dotted line marks the calculated shortest wall, and the magenta arrow indicates the polarity domain. Scale bar-5μm. Kolmogorov-Smirnov test comparing the Δθ°s distributions in the two ACD classes: p < 0.0001. H. Quantification of Δθ° during progenitor divisions in *basl*. Kolmogorov-Smirnov test with WT ACDs: p=0.0005. n = 94 cells. I. Examples of three early-lineage divisions in *basl* with associated Δθ°s. The black dotted line marks the predicted shortest wall. Scale bar-5μm. J. Representative images of a medial (left) and maximum projection (right) view of preprophase band (PPB) placement relative to the polarity domain in BRXL2p::BRXL2-YFP TMMp::mCherry-TUA5. Black arrows indicate the PPB. Scale bar-5μm. K. Reslice of the cell in (J) showing microtubule and BRXL2 distribution along the anticlinal wall. Black arrows indicate the PPB. Scale bar-5μm. L. Line scan along the cell cortex of the asymmetrically dividing cell shown in (J). The arrows indicate the position of the PPB. M. Microtubule distribution (gray lines) in PPB-forming cells (n=26 cells, average shown in black), aligned to the midpoints of the BRXL2 crescents (magenta line shows the mean ± standard deviation).

To interrogate how plant cells couple asymmetric divisions to identity specification, we monitored the cell division and differentiation dynamics of stomatal precursors in the *Arabidopsis* leaf epidermis (Fig. 1A). In this lineage, flexible asymmetric divisions in morphologically heterogenous early lineage cells create and pattern stomata (cellular valves that mediate plant-atmosphere gas exchange) by priming divergent developmental trajectories in the two daughters. The smaller meristemoid will eventually give rise to the paired guard cells that comprise a stoma, and the larger stomatal lineage ground cell (SLGC) will expand to become a pavement cell.

All asymmetric cell divisions within the lineage are preceded by the formation of a plasma membrane-associated polarity complex defined by BREAKING OF ASYMMETRY IN THE STOMATAL LINEAGE (BASL) (10) and BREVIS RADIX family (BRXf) (11) proteins (Fig. 1, A and B). Before mitosis, the BASL/BRXf crescent 1) recruits another polarly localized protein, POLAR (12), which in turn enriches GLYCOGEN SYNTHASE KINASE3 (GSK3)-like kinases at the cortex to promote asymmetric divisions (13), and 2) directs nuclear migration to bias the division site (14). After it is inherited by the SLGC through the asymmetric division, cortical BASL/BRXf enhances MITOGEN ACTIVATED PROTEIN KINASE (MAPK) signaling to suppress meristemoid identity (13, 15). Despite its central role in coordinating asymmetric division entry and daughter-cell identity post-division (16), how BASL/BRXf asymmetric inheritance is regulated is unknown (Fig. 1B).

We performed time-lapse imaging of developing cotyledons harboring markers for nuclei (R2D2 (17)), the plasma membrane (ML1p::mCherry-RCI2A) and the polarity crescent (BRXL2p::BRXL2-YFP). In agreement with previous observations, all asymmetric divisions resulted in singular inheritance of the BRXL2 crescent. Close analysis of these cells, however, revealed two asymmetric division subclasses that were defined by their division planes. The majority (73% of divisions) divided along the calculated shortest distance that intersected opposing cell walls at the site of the nucleus (small Δθ°, see Materials and Methods) (Fig.1, C to F), following the expectations set by the observed division planes in many plant cell types (18, 19). For this class of asymmetric divisions, polarity-directed nuclear migration (14) coupled with minimization of the division plane accurately predicted the final division site.

Division planes in the second class of asymmetric divisions (27% of divisions) deviated significantly from the calculated shortest wall (Fig. 1, D, E and G), suggesting that additional inputs control orientation of these early lineage divisions. Stomatal lineage divisions are not unique in breaking the shortest wall rule, but other cases during *Arabidopsis* development are related to broad, extrinsic influences such as tissue mechanics or hormone signaling (20, 21). Discrete, cell-autonomous mechanisms that tune division orientation have not been described. The morphological heterogeneity of stomatal precursors was well-represented within both asymmetric division subclasses (Fig. 1, F and G), suggesting that unique geometric features do not define asymmetric division subtypes. Instead, asymmetrically dividing cells specifically bypassed the calculated shortest division plane (large Δθ°) when that wall was predicted to intersect the plasma membrane within the cortical polarized site (Fig. 1G). Importantly, if only the non-polarized membrane was considered permissive for division plane placement, these asymmetric divisions continued to follow the shortest wall rule (fig. S1). This correlation suggested that polarized BASL/BRXf may be a cell-intrinsic cue capable of constraining potential orientations to control its own asymmetric inheritance.

Next, we tested whether cell polarity is necessary to stratify the two asymmetric division classes by tracking progenitor divisions in *basl* mutants (*basl* 35Sp::PIP2A-RFP ML1p::H2B-YFP). Loss of cellular polarity in *basl* collapsed the two asymmetric division classes into one (Fig. 1, H and I) that varied significantly from the total wild-type asymmetric divisions (Kolmogorov-Smirnov test, p=0.0005). Importantly, *basl* divisions did not differ significantly from wild-type asymmetric divisions without polarity conflict (p=0.1568), indicating that *basl* divisions follow the shortest wall rule. Therefore, the polarized BASL/BRXf domain is required to override default division patterns during formative asymmetric divisions.

## BASL influences preprophase band position

To determine the basis of this control, we examined the cortical microtubule structures that play essential roles during division orientation in plant cells (22). TAN1p::CFP-TAN1 foci, which mark the cortical division zone (23), never appear within the BASL/BRXf domain, suggesting that BASL/BRXf operate at an early step during division orientation (fig. S2). We found that the preprophase band of microtubules, which is the first marker of the eventual cortical division site, never formed within the polarity domain (Fig. 1, J to M). An analysis of the Δθ° between the preprophase band and calculated shortest wall showed a similar bifurcation of asymmetric divisions into two classes: the majority (66%) had preprophase bands that closely aligned to the predicted shortest wall while preprophase bands in the second class (34%) deviated significantly from the shortest distance (fig. S3). In this second class, 1) preprophase bands did not align with the predicted shortest wall when it bisected the polarity domain, and 2) polarity was required for preprophase band realignment away from the shortest wall (fig. S3). Together, these data indicated that the BASL/BRXf polarity crescent might orient divisions by controlling preprophase band placement.

**Figure 2.**
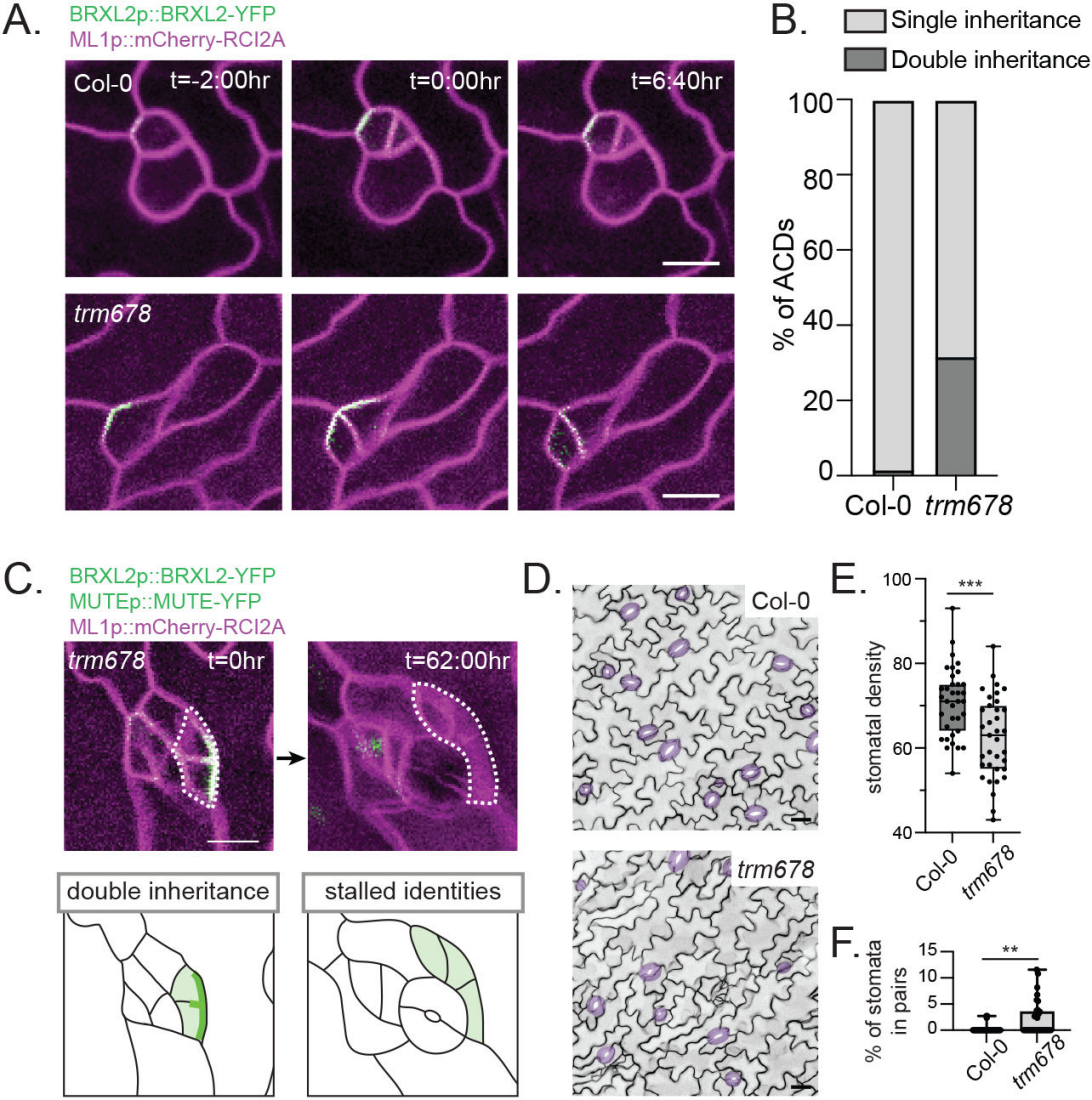
Singular inheritance of the BASL/BRXf polarity domain via preprophase band placement is required for stomatal patterning. A. Stills from representative time-lapse movies showing cell division orientation in Col-0 (top) and *trm678* (bottom), relative to the BRXL2-YFP-marked cortical polarity domain. Scale bars-10μm. B. Quantification of Col-0 (n=112) and *trm678* (n=116) asymmetric divisions (ACDs) with singular inheritance (BRXL2 inherited by only one daughter) or double inheritance (BRXL2 inherited by both daughter cells). C. Representative time-course images, with associated cartoons, showing an instance of double inheritance and cell fate stalling 62 hours later from a *trm678* seedling. Scale bar-25μm. D. Representative images of the epidermis of 7dpg Col-0 and *trm678* cotyledons expressing ML1p::mCherry-RCI2A. Stomata are pseudo-colored purple. Scale bars-25μm E. Quantification of stomatal densities (number of stomata per 581.82μm x 581.82μm area) in 7dpg Col-0 and *trm678* cotyledons (35 seedlings each). Unpaired t-test — p=0.0002. F. Quantification of the percent of paired stomata per 581.82μm x 581.82μm area in 7dpg Col-0 and *trm678* cotyledons (35 seedlings each). Unpaired t-test — p=0.0012.

To test this hypothesis, we generated lines to monitor BRXL2 inheritance in the *trm678* mutant (*trm678* BRXL2p::BRXL2-YFP ML1p::mCherry-RCI2A), which does not form preprophase bands (24). In contrast to wild-type asymmetric divisions, where BRXL2 was inherited by a single daughter cell, new cell walls frequently bisected the polarity site in *trm678* (32% of divisions) (Fig. 2, A and B), showing that the preprophase band is required to ensure complete inheritance of polarized BASL/BRXf. Next, we tracked cell fate outcomes following incorrect BASL/BRXf inheritance by monitoring progression through the stomatal lineage by tracking MUTE (25), a transcription factor that establishes the identity of the immediate stomatal guard cell precursor. *trm678* asymmetric divisions where BRXL2 was correctly inherited by a single daughter cell showed normal lineage progression; MUTE expression was detectable in the smaller cell after the division, and all tracked MUTE^+^ cells in *trm678* became paired guard cells (fig. S4). In contrast, *trm678* cells where cortical BRXL2 was bisected by the nascent division plane tended to generate daughters that 1) both inherited cortical BRXL2, 2) never transitioned to MUTE^+^ cells, and 3) failed to become pavement cells (Fig. 2C). In agreement with these tracking data, 7dpg *trm678* cotyledons had fewer stomata and a mispatterned epidermis (Fig. 2, D to F). We did not observe any alterations in the relationship between microtubules and BRXL2 in interphase *trm678* (fig. S4, D to F). Therefore, we conclude the preprophase band serves as an essential link between the polarity domain and division orientation to regulate stomatal identity.

## Cortical BASL domains are locally depleted of stable microtubules

**Figure 3.**
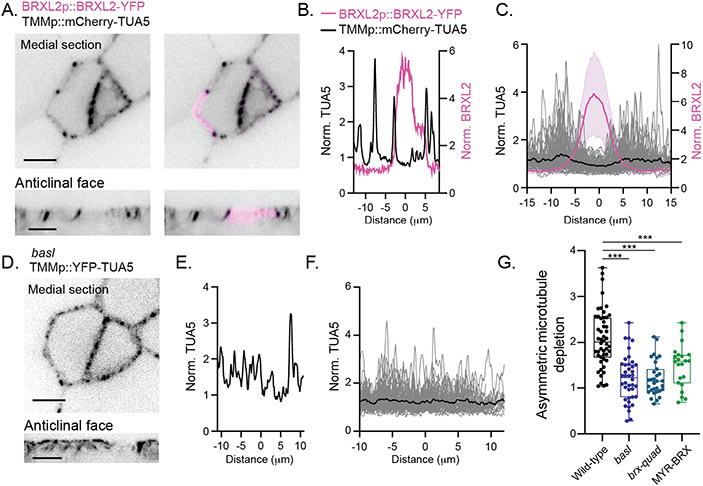
The BASL/BRXL2 domain locally depletes cortical microtubules from the plasma membrane. A. (Top) Representative medial section of a polarized stomatal lineage ground cell (SLGC) and associated meristemoid in BRXL2p::BRXL2-YFP TMMp::mCherry-TUA5. (Bottom) Reslice showing microtubule and BRXL2 distribution along the anticlinal face of the same cell. Scale bars-5μm. B. Line scan along the cell periphery of the SLGC shown in (A) showing the normalized BRXL2 and TUA5 fluorescence intensities. C. Microtubule distribution in SLGCs (gray lines) (n=72 cells), aligned to the midpoints of the BRXL2 crescents (magenta line corresponds to the mean signal ± standard deviation). The black line is the average of the plotted microtubule signals. D. (Top) Representative medial section of stomatal lineage cells in *basl* TMMp::YFP-TUA5. (Bottom) Reslice showing microtubule distribution along the anticlinal face of the same cell. Scale bars-5μm. E. Line scan along the cell cortex of the larger daughter cell in (D) showing the normalized TUA5 fluorescence intensity. F. Microtubule distribution in *basl* SLGCs (gray lines) (n=58 cells). G. Asymmetric microtubule depletion scores, calculated from integrated fluorescence intensities in polarized and unpolarized cortical domains (see Materials and Methods) in wild-type (n=50), *basl* (n=40), *brx-quad* (n=31), and BASLp::MYR-BRX (n=22) SLGCs. One-way ANOVA with Tukey’s post hoc test– ***-p < 0.0001.

How does the BASL/BRXf crescent influence preprophase band establishment? By creating a stomatal lineage-specific microtubule reporter line (TMMp::mCherry-TUA5), we could analyze microtubules and the BRXL2 polarity domain along anticlinal walls with high resolution (Fig. 3A). Unexpectedly, anticlinal microtubules were strongly depleted from the plasma membrane within the polarized domain, even in interphase SLGCs (Fig. 3, A to C). We confirmed that the same microtubule depletion zone occurred was observed when using a second polarity reporter, BASL, and in stomatal lineage cells of true leaves (fig. S5). POLAR, which shows overlapping but distinct localization from BASL/BRXf (12), co-localizes with microtubules outside the BASL/BRXL2 domain (fig. S5), indicating that microtubule depletion is correlated specifically with BASL/BRXf and is not a generalized activity of polarized proteins in the stomatal lineage.

BASL/BRXf could 1) locally deplete microtubules or 2) opportunistically polarize to already microtubule-poor regions. To distinguish between these possibilities, we performed two analyses. First, we examined cortical microtubule distribution before BASL polarization and found no microtubule-depleted region (fig. S6). Second, we compared microtubule distribution in wild-type and polarity-defective SLGCs and found that microtubule distribution was more homogenous in *basl and brx-quad* (11), which abrogate polarity, and in lines where addition of a myristoylation signal (BASLp::MYR-BRX-YFP (11)) renders BRX localization largely uniform at the cortex (Fig. 3, D to G, fig. S7). We also followed unmanipulated SLGCs as they lost polarized BASL/BRXf several hours after cell division. Our time course analysis showed that anticlinal microtubules reappeared within previously polarized regions in mature SLGCs (fig. S7D). From these data, we conclude that BASL/BRXf polarity creates and is required to maintain local microtubule loss at the plasma membrane.

Mutual inhibition by opposing plasma membrane-associated domains can drive polarization, as in the conserved PAR networks in animals (26) or a recently described polarity system in the monocot *B. distachyon* (27). Because our data raised the possibility that microtubules and cortical BASL/BRXf could operate in an analogous manner and inhibit the spread of each other, we tested whether altering microtubule distribution affected the stomatal lineage polarity domain. In agreement with previous results (28, 29), we found that microtubules are not necessary for the formation of a polarized BASL/BRXf domain (fig. S8A). However, quantification revealed a slight but significant spread of the polarity domain along the anticlinal wall in the absence of microtubules (fig. S8B). Short plasmolysis treatments, which dramatically disrupt cortical microtubule distribution, similarly altered polarity boundaries and polarity domain size without complete depolarization (fig. S8C). Therefore, microtubules shape BASL/BRXf domain boundaries although they are dispensable for polarity itself. Intriguingly, this interaction shares striking similarities with microtubule-mediated sculpting of ROP GTPase domains in non-dividing cells of the xylem and in trichomes (30-32).

## Microtubule dynamics are locally altered within polar domains

How do cortical BASL/BRXf locally deplete cortical microtubules? Owing to the technical challenges associated with monitoring dynamic microtubule behavior along the anticlinal wall of meristemoids, we created a heterologous system where we could track microtubule dynamics co-incident with the BASL polarity domain. By introgressing a ubiquitous microtubule reporter (35Sp::mCherry-TUA5) into a line expressing a hyperactive version of BASL capable of rescuing the *basl* phenotype (35Sp::GFP-BASL-IC, hereafter referred to as BASL^ectopic^ (10)), we could monitor microtubule organization relative to BASL in the hypocotyl epidermis. BASL^ectopic^ locally depleted microtubules along anticlinal walls in the hypocotyl epidermis as in the stomatal lineage (fig. S9), demonstrating that this ectopic system recapitulates the molecular interactions found in the leaf epidermis.

**Figure 4.**
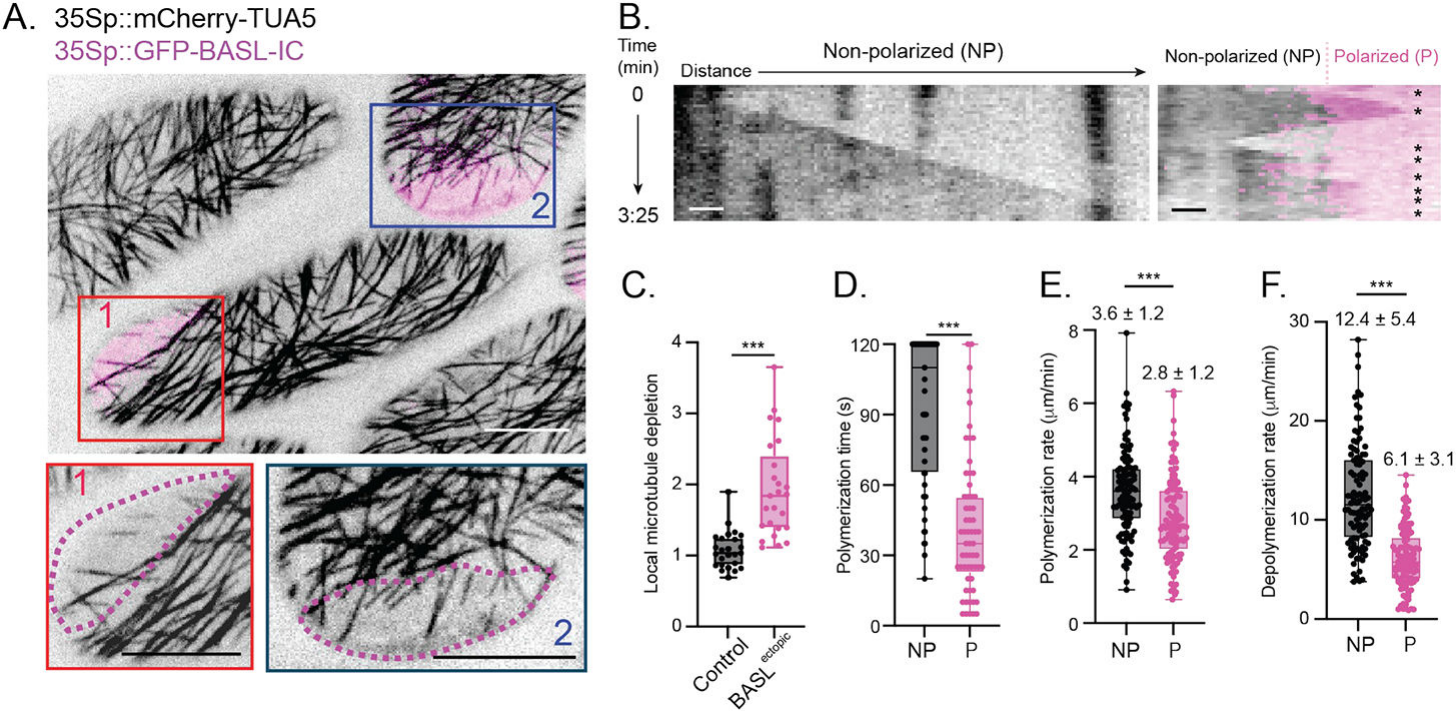
Polarized BASL destabilizes microtubule plus-ends to locally bias array organization. A. Representative images of the apical surfaces in epidermal cells of 35Sp::GFP-BASL-IC 35Sp::mCherry-TUA5 hypocotyls. The boxed regions below highlight the microtubule organization within the polarized domains of two cells. Scale bars-10μm. B. Kymographs showing microtubule plus-end dynamics within a non-polarized region (left) and an ectopic polarity domain (right). Asterisks indicate microtubule catastrophes and rescues. Scale bar-1μm. C. Local microtubule depletion within apical BASL^ectopic^ domains and comparably sized random regions in control hypocotyls. The local microtubule depletion was derived using 35Sp::mCherry-TUA5 fluorescence (see Materials and Methods). n=25 hypocotyl cells for each. Unpaired t-test — p < 0.0001. D-F. Quantification of microtubule plus-end dynamics within non-polarized (NP) and polarized (P) apical domains. Growth persistence (D), polymerization rate (E) and depolymerization rate (F) were quantified. The numbers above the box-and-whisker plots are mean values ± standard deviation. For all comparisons, unpaired t-tests were used — p < 0.0001.

BASL^ectopic^ domains extended onto the apical surfaces of hypocotyl epidermal cells and locally depleted cortical microtubules (Fig. 4, A and C), allowing us to observe the BASL-mediated effects on microtubules with precision not possible within the stomatal lineage. Increased microtubule severing has been identified as a key reorganizer of cortical microtubule arrays during several developmental transitions (33, 34). However, as severing preferentially occurs at microtubule crossover sites (35, 36) and there were few crossovers in microtubule-depleted BASL^ectopic^ regions, severing was largely suppressed within BASL^ectopic^ domains (fig. S10, A and B). Tracking of microtubule minus ends within BASL^ectopic^ also indicated that local microtubule depletion was not due to decreased minus-end stability (fig. S10C). Instead, we found that BASL^ectopic^ had two effects on microtubule plus-ends. First, plus-end polymerization and depolymerization rates were significantly suppressed within BASL^ectopic^ (Fig. 4, E and F, fig. S10D). Second, we observed that microtubule plus-ends rapidly underwent catastrophe upon entering the BASL^ectopic^ domain (Fig. 4, B and D). Increased catastrophe rates often led to complete loss of the microtubule, reestablishing the microtubule depletion zone.

To validate that our BASL^ectopic^ findings in the hypocotyl reflect BASL-microtubule interactions within the stomatal lineage, we used two independent approaches. First, we performed time-lapse imaging of stomatal progenitors in BRXL2p::BRXL2-YFP TMMp::mCherry-TUA5 seedlings and observed transient microtubules within the polarity domain that were rapidly depolymerized (fig. S11A). Second, to monitor growing plus ends with higher precision along the anticlinal wall, we generated a stomatal lineage-specific END BINDING PROTEIN 1b (EB1b) reporter (TMMp::EB1b-mCherry) and introgressed it into the BRXL2 reporter line. Fewer EB1b puncta were observed within the native polarity domain in SLGCs than in non-polarized regions of the same cells (fig. S11, B to D). Therefore, our analyses of microtubule dynamics in native and heterologous systems reveal that the BASL/BRXf domain destabilizes microtubule plus ends to locally deplete them from the polarized region.

## Discussion

As encoders of spatial information, polarity domains are central regulators of asymmetric cell division in diverse plant tissues (37-40). Here, we provide evidence that the BASL/BRXf polarity domain robustly serves dual functions to orient asymmetric divisions and specify cell identity by controlling its own inheritance via negative interactions with the microtubule cytoskeleton. Polarity-microtubule interactions now emerge as a common theme to guide asymmetric inheritance of fate regulators during both metazoan and plant asymmetric divisions, albeit through fundamentally different mechanisms (fig. S12). In the canonical asymmetric cell division pathway in animal cells, the cortical polarity domain is responsible for 1) localizing fate determinants to one pole and 2) subsequently directing the division angle to ensure their singular and asymmetric inheritance by exerting pulling forces on astral microtubules (41, 42). The model we advance here differs in several significant ways. First, the proposed mechanism utilizes core plant-specific mitotic structures without the need to invoke a role for astral microtubules, which are absent in plant spindles. Second, rather than ensuring its singular inheritance by precisely specifying the ultimate division plane, BASL/BRXf renders a region of the membrane unavailable as the division site. Third, while this mechanism has an identical outcome—asymmetric inheritance of key fate regulators—it is uniquely suited for a morphologically heterogenous population that, nonetheless, must robustly couple asymmetric division orientation with subsequent daughter cell identities.

How does BASL-mediated polarity modulate microtubule dynamics? BASL and BRXf proteins both contain large, disordered regions (43), leading us to favor two general models. In the first, BASL and BRXf scaffold effectors that 1) directly affect plus-end kinetics and 2) potentially bind along the microtubule lattice to impact depolymerization rates. From our analyses in this work, we anticipate that such microtubule-associated effectors would be expressed throughout the cell cycle and in multiple tissues, which has complicated our ongoing efforts to identify them. In the second model, which does not invoke additional downstream factors, polarization via phase-separation could tune the local physical properties of the membrane-adjacent cytoplasm; such a mechanism can modulate microtubule dynamics in yeast (44), and might be hinted at by the dampening of MT dynamics at the polarity zone (fig. S10D).

Of the documented, polarity-mediated asymmetric divisions in plants, those that violate the shortest wall rule, such as those in the early *Arabidopsis* embryo or subsidiary mother cell divisions in *Zea mays*, may be the closest corollaries to the system presented here. While BASL is both eudicot (43) and stomatal lineage specific, BRX family proteins participate in additional cellular decisions in *Arabidopsis* and are much more deeply conserved in the green lineage (45), hinting that other tissue-specific regulators could provide context specificity to a common polarity core. Further analysis of polarity will help clarify whether this mechanism is shared across plant tissues and species or whether it has evolved for the challenges associated with flexible patterning in the eudicot stomatal lineage.

## Supplementary Material

1
