# Supplementary material for: Cortical polarity ensures its own asymmetric inheritance in the stomatal lineage to pattern the leaf surface
Source: Science. Author manuscript; Available in PMC 2023 Jul 7. (PMC10328556; doi:10.1126/science.add6162)
Supplement: 1 [file NIHMS1910966-supplement-1.pdf]

Science
AAAS

Supplementary Materials for

Cortical polarity ensures its own asymmetric inheritance in the stomatal lineage to pattern the leaf surface

Andrew Muroyama*, Yan Gong, Kensington S. Hartman, Dominique Bergmann*

*Correspondence to: bergmann@stanford.edu and amuroyama@ucsd.edu

This PDF file includes:

Materials and Methods
Figs. S1 to S12

Materials and Methods

Plant material and growth

Arabidopsis thaliana seeds were sterilized in 20% bleach with 0.1% Tween-20 for 10 minutes, washed three times with dH_2O, and plated onto ½ MS (Murashige & Skoog media) plates with 0.5% sucrose. Plates were stratified in the dark for at least two days at 4°C. Seedlings were grown on plates under long day conditions (16hr light/8hr dark) at 22°C.

All *Arabidopsis thaliana* lines used in this study were in the Col-0 background. The same null allele of *BASL* (*basl-2*) (*10*) was used for all analyses of BASL function and is referred to in the text as *basl*. The quadruple T-DNA mutant of *brx, brxl1, brxl2, brxl3* is referred to as *brx-quad* (*11*). Previously reported *Arabidopsis* lines used in this study include: R2D2 BRXL2p::BRXL2-YFP ML1p::mCherry-RCI2A (*14*), *basl-2* ML1p::H2B-YFP 35Sp::PIP2A-RFP (*14*), *basl-2* (WiscDsLox264F02) (*10*), *brx-quad (11)*, BASLp::MYR-BRX-YFP (*11*), BRXL2p::BRXL2-YFP (*11*), 35Sp::GFP-BASL-IC (*10*), 35Sp::mCherry-TUA5 (*46*), BASLp::YFP-BASL TMMp::mCherry-TUA5 (*14*), *trm678 (24)*, BRXL2p::BRXL2-YFP 35Sp::mCherry-TUA5 (*29*), and ML1p::mCherry-RCI2A (*47*). TAN1p::CFP-TAN1 seeds were a kind gift from Dr. Carolyn Rasmussen (UC Riverside) (*48*).

BRXL2p::BRXL2-YFP TMMp::mCherry-TUA5, BASLp::MYR-BRX-YFP TMMp::mCherry-TUA5, and POLARp::POLAR-YFP TMMp::mCherry-TUA5 were generated by introducing TMMp::mCherry-TUA5 R4pGWB601 (*14*) into the respective parental lines via agrobacterium-mediated transformation. T1s were selected on ½ MS plates containing 50µM phosphinothricin. To generate *basl-2* TMMp::YFP-TUA5 and *brx-quad* TMMp::YFP-TUA5. TMMp::YFP-TUA5 R4pGWB501 was introduced into the parental lines using agrobacterium-mediated transformation. T1 seeds were selected on ½ MS plates with 15µg/mL hygromycin. BRXL2p::BRXL2-YFP (*11*), ML1p::mCherry-RCI2A (*47*), TMMp::mCherry-TUA5 R4pGWB601 (*14*) and MUTEp::MUTE-YFP (*49*) were individually introduced into *trm678* by agrobacterium-mediated transformation. Combinatorial reporters in the *trm678* background were generated by crossing. 35Sp::GFP-BASL-IC 35Sp::mCherry-TUA5 plants were generated by crossing. TAN1p::CFP-TAN1 BRXL2p::BRXL2-YFP TMMp::mCherry-TUA5 plants were generated by crossing TAN1p::CFP-TAN1 with BRXL2p::BRXL2-YFP TMMp::mCherry-TUA5.

Cloning and agrobacterium-mediated transformation

TMMp::YFP-TUA5 R4pGWB501 was generated by Gateway cloning (Invitrogen). The YFP sequence was amplified with the following primers: YFP F- 5'-gcggccgcatggtgagcaagggcgaggag-3' and YFP R - 5'-gcggccgccttgtacagctcgtccatgc-3' and subcloned into TUA5 pENTR D-TOPO (*14*) using NotI sites to create YFP-TUA5 pENTR D-TOPO. The TMM promoter in pDONR P4-P1R (540 bp upstream of *TMM*), YFP-TUA5 pENTR D-TOPO, and R4pGWB501 (*50*) were recombined using LR Clonase II (Invitrogen) to create TMMp::YFP-TUA5 R4pGWB501. The construct was confirmed by sequencing. Constructs that

were used for plant transformation were introduced into the GV3101 strain of agrobacterium via electroporation.

Drug and plasmolysis treatment (related to fig. S8)

For oryzalin experiments, 3dpg BRXL2p::BRXL2-YFP TMMp::mCherry-TUA5 seedlings were incubated in liquid ½ MS + 0.75% sucrose with 20μM oryzalin (Sigma-Aldrich CAS Number 19044-88-3) or DMSO for 2 hours. After the incubation, seedlings were mounted on slides for imaging in their respective solutions. For plasmolysis experiments, BRXL2p::BRXL2-YFP TMMp::mCherry-TUA5 seedlings were briefly treated (<10min) in 0.8M mannitol before mounting for imaging.

Image acquisition

Four microscope setups were utilized for these studies. For time-lapse experiments of division orientation in wild-type (Fig. 1, D to G, fig. S1A), *basl* (Fig. 1, H and I), and *trm678* (Fig. 2, A to C) and the analysis of fate outcomes in *trm678* (fig. S4C), seedlings were mounted in ½ MS + 0.75% sucrose in a custom-fabricated imaging chamber (*51*). The chamber was connected to a peristaltic pump set to a flow rate of 2 mL/hr. Time-lapse movies were acquired on a Leica SP5 with 25x 0.95 NA and 40x 1.1 NA water immersion objectives and HyD detectors using LAS X software. For wild-type and *basl* time-lapse movies, images were acquired every 30 minutes. For *trm678* time-lapse movies, images were acquired every 40 minutes.

Images of the cotyledon epidermis in Col-0 ML1p::mCherry-RCI2A and *trm678* ML1p::mCherry-RCI2A (fig. S4, A and B) and BRXL2p::BRXL2-YFP ML1p::mCherry-RCI2A (Fig. 1A) were acquired on a Leica SP5 with a 25x 0.95 NA water immersion objective.

Phenotypic analysis of the *trm678* mutants and Col-0 controls (Fig. 3, D to F) was performed using a Leica Stellaris 5 with HyD S detectors and a PL APO 20x 0.75 NA objective using LAS X software. Images of polarized divisions in TAN1p::CFP-TAN1 BRXL2p::BRXL2-YFP TMMp::mCherry-TUA5 (fig. S2) were acquired on the same Leica Stellaris system with an APO 63x 1.20 NA water objective.

For the analyses of microtubule/preprophase band distribution in wild-type Col-0 (Fig. 1, J to M, Fig. 3, A to C and G, fig. S1B, fig. S3, fig. S5, A to F, fig. S7E, fig. S11A), microtubule distribution in *basl* (Fig. 3, D to G), microtubule distribution in *brx-quad* (Fig. 3G, fig. S7, A to B), microtubule distribution in MYR-BRX (Fig. 3G, fig. S7, C to D), microtubule distribution on the apical surfaces of BASL^{ectopic} (Fig. 4, A and C), oryzalin treatments (fig. S8A), and plasmolysis experiments (fig. S8C), a spinning disk confocal microscope with a DMI6000 (Leica) stand, Evolve EMCCD (Photometrics) camera, SlideBook software (3i) and 100x 1.4 NA objective was used.

For the analyses of microtubule distribution in *trm678* (fig. S4, D to F), POLAR localization with cortical microtubules in Col-0 (fig. S5, G to H), microtubule organization before mitotic onset in BASLp::YFP-BASL TMMp::mCherry-TUA5 (S6), microtubule organization and behavior in BASL^{ectopic} cells (Fig. 4, B and D to F, fig. S9 and fig. S10), and EB1b dynamics (fig.

S11, B to D), images were acquired using a SR HP APO TIRF 100x 1.49 NA objective on a Nikon Eclipse Ti2-E microscope with Prime 95B sCMOS (Photometrics) camera and NIS-Elements software.

Cortical fluorescence intensity measurements and background normalization (related to Fig. 1, L and M, Fig. 3, B to C and E to F, fig. S4F, fig. S5, fig. S6, fig. S7, fig. S9)

Fluorescence intensity profiles along the cortex were generated from a 3-pixel wide line in FIJI that traced the cell periphery. Importantly, fluorescence intensity profiles along the SLGC cortex exclude the membrane that is shared with the neighboring meristemoid. This membrane was excluded from analysis because the TMM promoter drives reporter expression in both SLGCs and meristemoids. As such, TUA5 signal along that membrane could not be conclusively attributed to cortical microtubules in the SLGC or meristemoid owing to the close juxtaposition of neighboring membranes.

BASL, BRXL2, and TUA5 signals were normalized to show fold-change over background. To do this, five separate regions were chosen at the cell cortex to serve as background. For BASL and BRXL2 signals, these were cortical regions that were not in the polar domain. For TUA5, these were cortical regions with cytoplasmic TUA5 signal but no detectable puncta (microtubules). The mean fluorescence intensity in these five background regions were calculated in FIJI and averaged to generate a single background value for that cell. Normalization was performed by dividing the fluorescence signal at each position along the line scan by the background value.

For wild-type polarized cells, line scans were aligned relative to the midpoint of the polarity domains. For *basl*, *brx-quad*, and MYR-BRX, which do not have a polar domain to serve as a reference alignment landmark, line scans were aligned relative to the membrane opposite the newly created meristemoid, as in (*14*).

Quantification of stomatal phenotypes in *trm678* (related to Fig. 2, D to F)

The stomatal phenotypes in *trm678* and paired wild-type Col-0 controls were determined from 7dpg seedlings. Stomatal densities were calculated by counting the number of stomata in a 581.82μm x 581.82μm area for one cotyledon per seedling. Stomatal pairs were quantified using the same images and are represented as the percent of total stomata that are in pairs.

Quantification of the predicted shortest wall during asymmetric division (related to Fig. 1, D to I, fig. S1A and fig. S3):

To calculate the shortest wall that could be created during asymmetric divisions, xy coordinates defining 1) the cellular outlines and 2) the nuclear centroids were manually extracted in FIJI from the frame immediately before cell division. Coordinates were imported into MATLAB (version 2021b), plotted, and used to calculate the minimal distance that bisects the cell boundary through the nuclear centroid using straight line distances extracted from calculated polar plots. The calculated shortest wall was plotted on the cell boundary, and each cell was individually inspected and validated.

The angle of the actual division plane was determined in FIJI from the frame immediately after cytokinesis completion. $\Delta\theta°$ is, therefore, the difference between the actual division plane and the calculated shortest wall.

A very similar approach was used to calculated $\Delta\theta°$ between the preprophase band and predicted shortest wall. In this case, however, the analyses were conducted on images taken at a single time point (cells with mature PPBs) instead of from time-lapse data. The xy coordinates for the cell outlines and nuclear centroids were extracted from images in FIJI and used to calculate the shortest wall in MATLAB. PPB angles were determined from the same images in FIJI.

<u>Quantification of asymmetric microtubule depletion</u> (related to Fig. 3G):

To calculate microtubule distribution in wild-type Col-0 SLGCs, the fluorescence intensities for both BRXL2p::BRXL2-YFP and TMMp::mCherry-TUA5 were measured along the cell cortex by generating line scans in FIJI. The polarized region of the cortex was determined using the outer bounds of the BRXL2 signal. The TUA5 fluorescence intensity along the cortex was plotted in MATLAB. The integrated area under the normalized microtubule fluorescence signal was calculated in MATLAB using trapezoidal numerical integration. The "Asymmetric microtubule depletion" was calculated by dividing the integrated intensity of the periphery outside the BRXL2 signal by the integrated intensity within the BRXL2 domain. To calculate the "Asymmetric microtubule depletion" for *basl*, *brx-quad*, and BASLp::MYR-BRX-YFP, microtubule intensity profiles were created in FIJI as in wild-type SLGCs. For each profile, the integrated area under a random region of the cortex ~10μm in length (comparable in size to the average BRXL2 domain in wild-type cells) was used to generate the "Asymmetric microtubule depletion" score (random region/rest of cortex).

<u>Quantification of local microtubule depletion in BASL^{ectopic}</u> (related to Fig. 4C):

To calculate microtubule occupancy within control (35Sp::mCherry-TUA5) and BASL^{ectopic} (35Sp::mCherry-TUA5 35Sp::GFP-BASL-IC), single optical sections along the apical surfaces of hypocotyl epidermal cells were used. For BASL^{ectopic}, the mean TUA5 fluorescence intensities within and outside the BASL domain were calculated in FIJI and normalized to the areas of their respective regions. To calculate "Local microtubule depletion," the normalized microtubule intensity outside the polar domain was divided by the normalized microtubule intensity within the domain. To calculate a "Local microtubule depletion" score for control cells, a random region of the apical surface of comparable size to the BASL^{ectopic} domains was specified and calculations were carried out in the same manner.

<u>Quantification of microtubule dynamics </u>(related to Fig. 4, D to F, fig. S10):

To quantify microtubule polymerization time (Fig. 4D), two-minute-long time-lapse movies (5 second intervals) were captured on a Nikon Eclipse Ti2-E microscope. Only individual microtubules where the plus-end could be definitively tracked for the length of the movie were used for quantifications. To calculate microtubule polymerization and depolymerization rates (Fig.

4, E and F), kymographs were manually generated for individual apical microtubules and were used to calculate the polymerization and depolymerization rates. To quantify severing frequencies (fig. S10A and B), the number of severing events per 100μm^2/min were counted in control (35Sp::mCherry-TUA5) cells and both polarized and non-polarized regions of $BASL^{ectopic}$ cells.

Quantification of EB1b density (related to fig. S11):

To calculate EB1b density, one-minute-long time-lapse movies (5 second intervals) of BRXL2p::BRXL2-YFP TMMp::EB1b-mCherry were acquired at mid-cell height on a Nikon Eclipse Ti2-E microscope. Kymographs showing EB1 puncta and BRXL2 along the cell cortex were generated by manual annotation of the cell periphery in FIJI. Background in the kymographs was reduced using "Despeckle," and the total number of EB1b puncta along the cortex over the one minute was determined using "Find Maxima" in FIJI. To determine the EB1b density within the polar domain, the BRXL2 signal in the kymograph was thresholded to specify the polarized region. The "Find Maxima" command in FIJI was used to mark those EB1b puncta specifically within the threshold region. All EB1b puncta that were identified with this method were manually inspected for validity. EB1b density was calculated as the number of EB1b puncta per 10μm along the cell cortex per minute.

Software and statistics

See the Image Acquisition section for information about software that was used to acquire imaging data. All image analysis was done with FIJI. MATLAB (version 2021b) was utilized to generate the polar histograms in Figure 1, fig. S1, and fig. S3 and to calculate the "Asymmetric microtubule depletion" score (Fig. 3G). All other graphs were generated in GraphPad Prism 9. All statistical analyses were performed using GraphPad Prism 9. For comparisons of wild-type divisions and preprophase bands to the calculated shortest wall, the Kolmogorov-Smirnov test was used. For comparisons between two conditions, unpaired t-tests were used except for the EB1b density analysis (fig. S11), where a paired t-test was used. For the analysis of three of more conditions, a one-way ANOVA with Tukey's multiple comparisons was used.

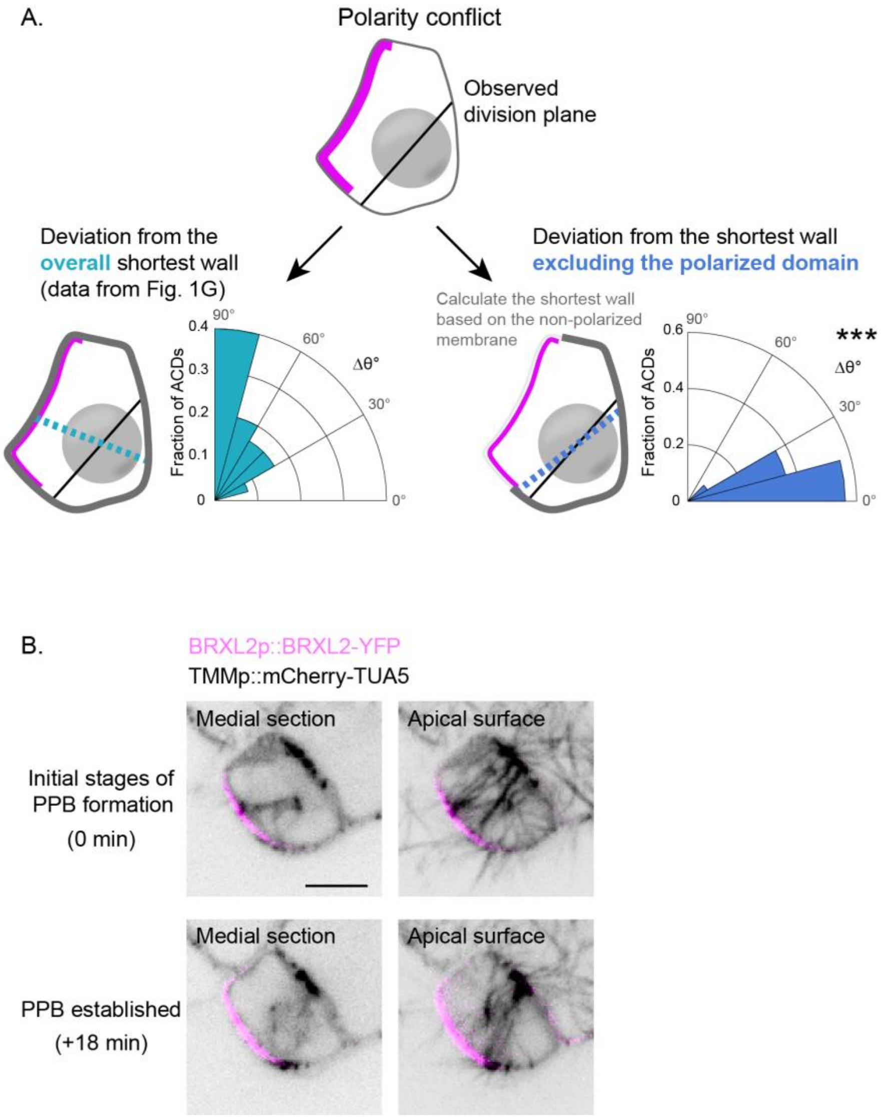

Fig. S1. Asymmetrically dividing cells build the shortest wall excluding the polarized membrane.

A. Comparison of division patterns in "polarity conflict" cells. On the left are the same data from Fig. 1G (n=25 cells), showing that the actual division angle deviates significantly from the predicted shortest wall in ACDs where the predicted shortest wall intersects the polarized membrane. On the right, we performed an additional analysis of the same cells where we calculated the shortest wall excluding the polarized membrane from the calculation. The real division plane closely matched the shortest available wall excluding the polarized membrane. This re-analysis indicated that "polarity conflict" cells continue to follow the shortest wall rule but build the shortest wall available without using the non-polarized membrane. Kolmogorov-Smirnov test, $p<0.001$.

B. Example of asymmetrically dividing cell during PPB establishment, showing how the PPB forms outside the polar BRXL2 domain (magenta). Scale bar-5μm.

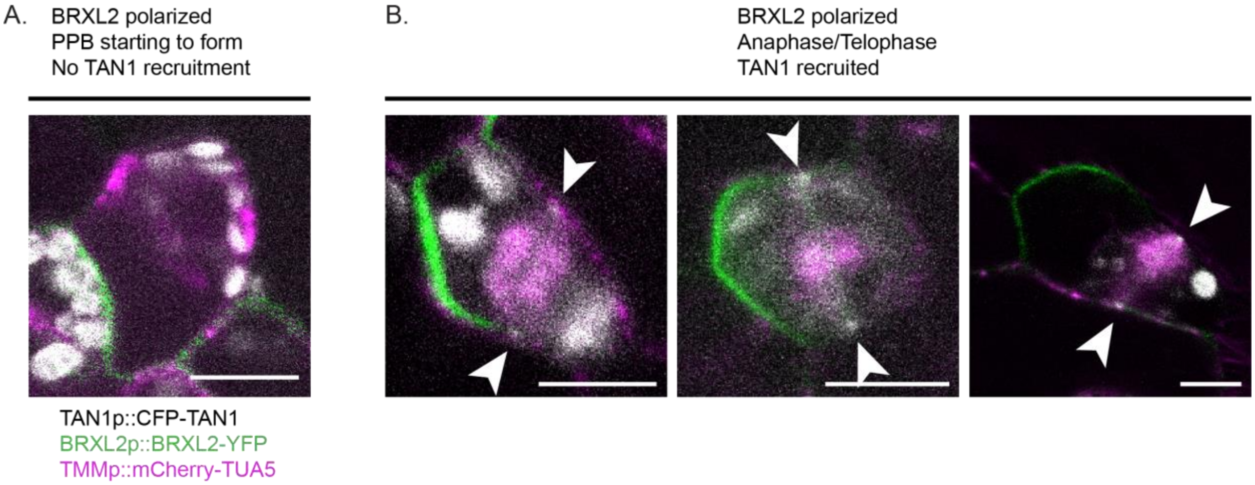

Fig. S2. TAN1 foci are found outside the polar domain.

A. BRXL2 (green) polarizes before TAN1 (white) recruitment to the cortical division zone. The cell shown here is in the process of PPB establishment (TUA5, magenta). Scale bar-5μm.

B. TAN1 recruitment is evident in anaphase and telophase, and TAN1 foci (white arrows) are always found outside of the polar domain. Scale bar-5μm.
Note that the autofluorescence from the chloroplasts appears in the same channel as TAN1p::CFP-TAN1. Based on previous analyses of this reporter in the root, only cortical foci were treated as bona fide TAN1 signal.

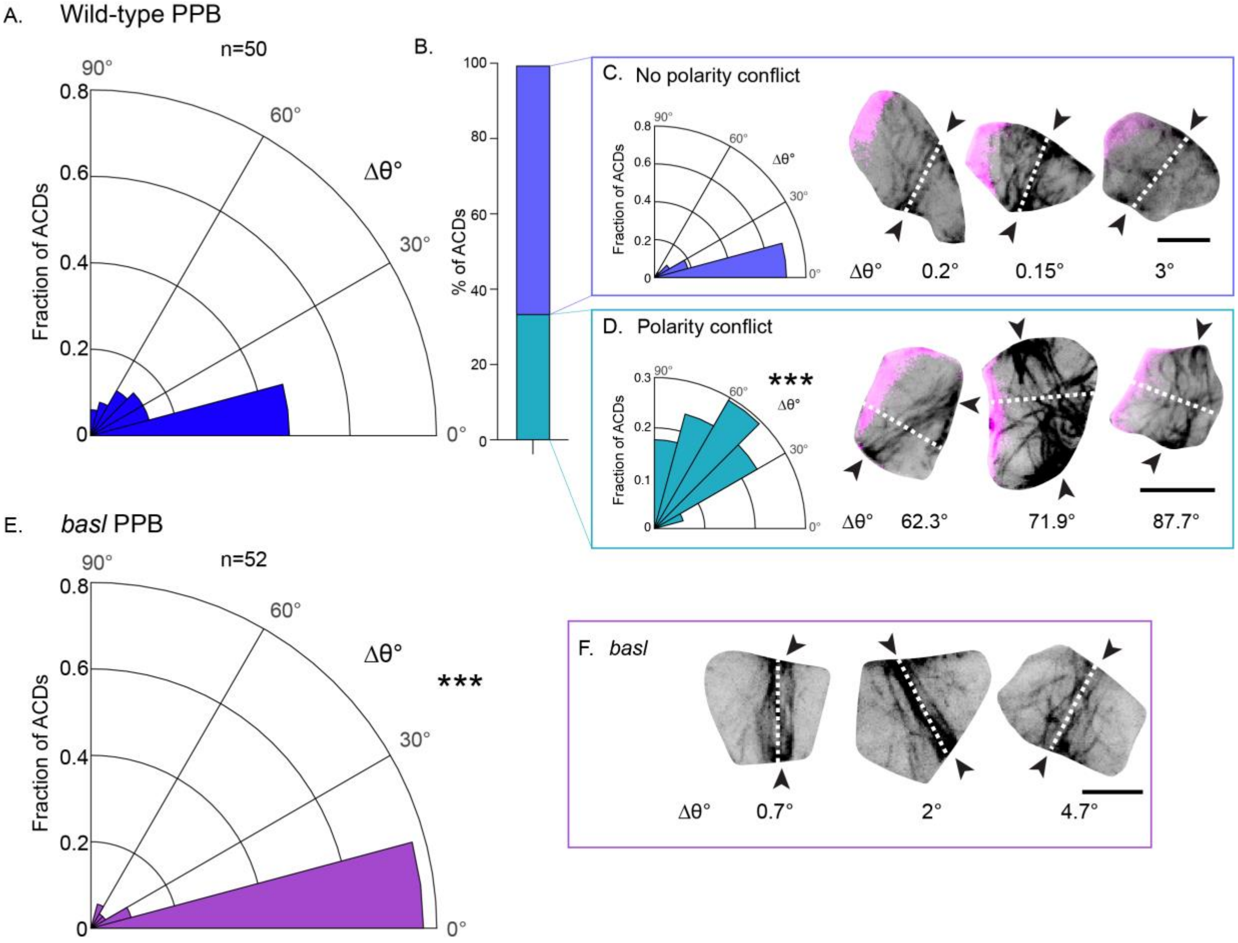

Fig. S3. The preprophase band avoids the overall shortest wall when there is a polarity conflict.

A. Quantification of Δθ° (the angle between the predicted shortest wall and the preprophase band) during wild-type ACDs. n = 50 cells.

B. The percentage of total ACDs where the position of the polarity domain did or did not conflict with the predicted shortest wall.

C-D. (Left) Distribution of Δθ° in ACDs where the predicted shortest wall did (D) or did not (C) conflict with the polarity domain. (Right) Three examples of preprophase bands visualized by TMMp::mCherry-TUA5 in cells expressing polarity marker BRXL2p::BRXL2-YFP (pink) in the respective ACD classes with associated Δθ°s. The dotted white lines mark the predicted shortest wall. Black arrows indicate the position of the PPB. Kolmogorov-Smirnov test comparing the two classes of ACDs: $p<0.0001$. Scale bar-5μm.

E. Quantification of Δθ° during progenitor divisions in *basl*. Kolmogorov-Smirnov test with WT ACDs: $p=0.0009$. n = 52 cells.

F. Three examples of early progenitor divisions in *basl* with associated Δθ°s from *basl* TMMp::YFP-TUA5. The dotted white line indicates the predicted shortest wall. Black arrows indicate the position of the PPB. Scale bar-5μm.

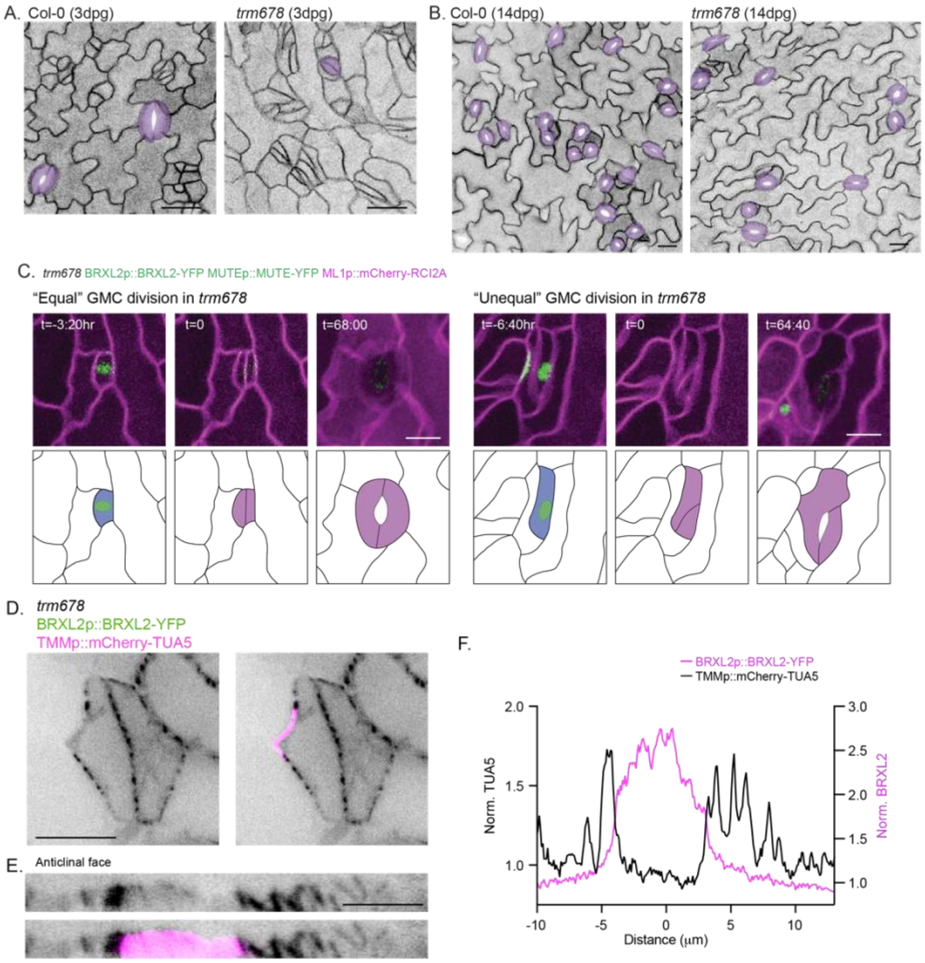

Fig. S4. Additional characterization of the *trm678* phenotype in developing cotyledons.

A. Representative images of 3dpg Col-0 (left) and *trm678* (right) cotyledons. Plasma membranes are visualized by ML1p::mCherry-RCI2A. Stomata are pseudo-colored purple. Scale bar-25μm.
B. Representative images of 14dpg Col-0 (left) and *trm678* (right) cotyledons. Plasma membranes are visualized by ML1p::mCherry-RCI2A. Stomata are pseudo-colored purple. Scale bar-25μm.
C. Representative stills, with associated cartoons, from a time-course analysis that revealed that 145/145 MUTE-expressing cells (green nuclei) that underwent division created paired guard cells. Even "unequal" GMC divisions yield two GCs capable of creating a stomatal pore. Scale bars-10μm.
D. Representative optical section at mid-cell height of a polarized SLGC and associated meristemoid (both in interphase) in *trm678* BRXL2p::BRXL2-YFP TMMp::mCherry-TUA5. Scale bar-10μm.
E. Reslice showing microtubule and BRXL2 distribution along the anticlinal face of the same cell. Scale bar-10μm.
F. Line scan showing relationship between anticlinal microtubules (TMMp::mCherry-TUA5) and BRXL2 (BRXL2p::BRXL2-YFP) in *trm678*.

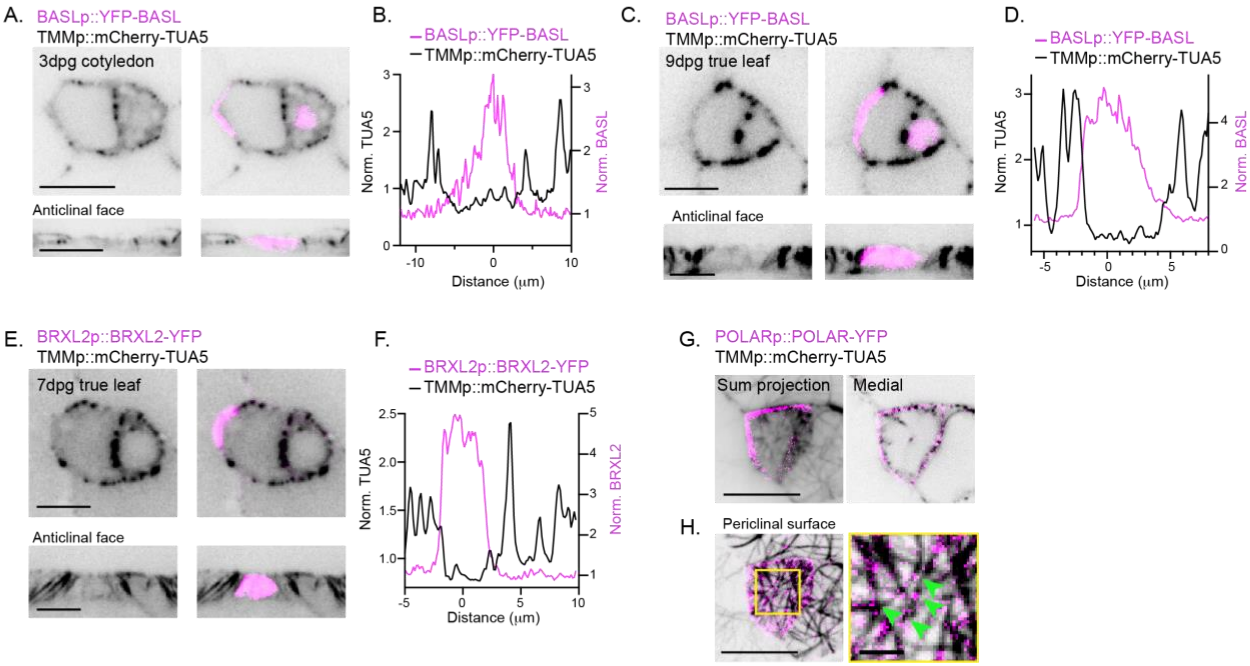

Fig. S5. BRXL2 and BASL, but not POLAR, mark microtubule-depleted regions in cotyledons and true leaves.

A. (Top) Representative medial section of a polarized SLGC and associated meristemoid in a 3dpg BASLp::YFP-BASL TMMp::mCherry-TUA5 cotyledon. (Bottom) Reslice showing microtubule and BASL distribution along the anticlinal face of the same cell. Image is representative of 116 examined cells. Scale bars-5μm.
B. Line scan along the cell cortex of the SLGC shown in (A).
C. (Top) Representative medial section of a polarized SLGC and associated meristemoid in a 9dpg BASLp::YFP-BASL TMMp::mCherry-TUA5 true leaf. (Bottom) Reslice showing microtubule and BASL distribution along the anticlinal face of the same cell. Image is representative of 51 examined cells. Scale bars-5μm.
D. Line scan along the cell cortex of the SLGC shown in (C).
E. (Top) Representative medial section of a polarized SLGC and associated meristemoid in a 7dpg BRXL2p::BRXL2-YFP TMMp::mCherry-TUA5 true leaf. (Bottom) Reslice showing microtubule and BRXL2 distribution along the anticlinal face of the same cell. Image is representative of 197 examined cells. Scale bars-5μm.
F. Line scan along the cell cortex of the SLGC shown in (E).
G. Microtubule distribution relative to POLAR in a 3dpg POLARp::POLAR-YFP TMMp::mCherry-TUA5 cotyledon. (Left) Sum projection of the cell volume. (Right) Medial section. Image is representative of 74 examined cells. Scale bar-10μm.
H. (Left) Periclinal surface of POLARp::POLAR-YFP TMMp::mCherry-TUA5 cell shown in (G). Scale bar-5μm. (Right) Zoomed region of the periclinal surface shows co-localization between POLAR foci and microtubules. The green arrows indicate sites where POLAR colocalizes with microtubules. Scale bar-2μm

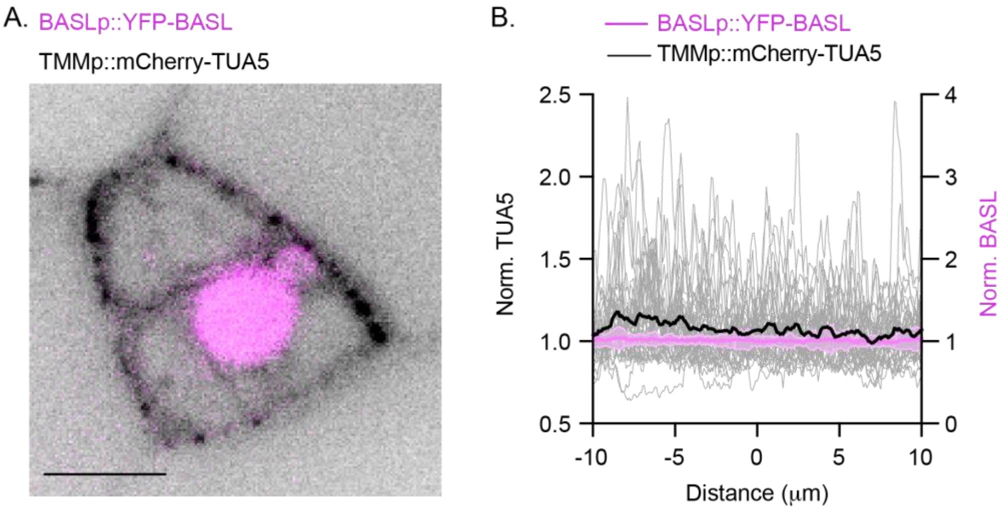

Fig. S6. Microtubule distribution before polarization.

A. Representative medial section of a meristemoid expressing BASL (magenta) at a stage in the cell cycle preceding BASL cortical polarization in a 3dpg BASLp::YFP-BASL TMMp::mCherry-TUA5 cotyledon. Scale bar-5μm.

B. Microtubule distribution (gray lines) in pre-polarized cells (n=38 cells). The black line shows the average microtubule signal and the magenta line shows the average cortical BASL signal (mean ± standard deviation).

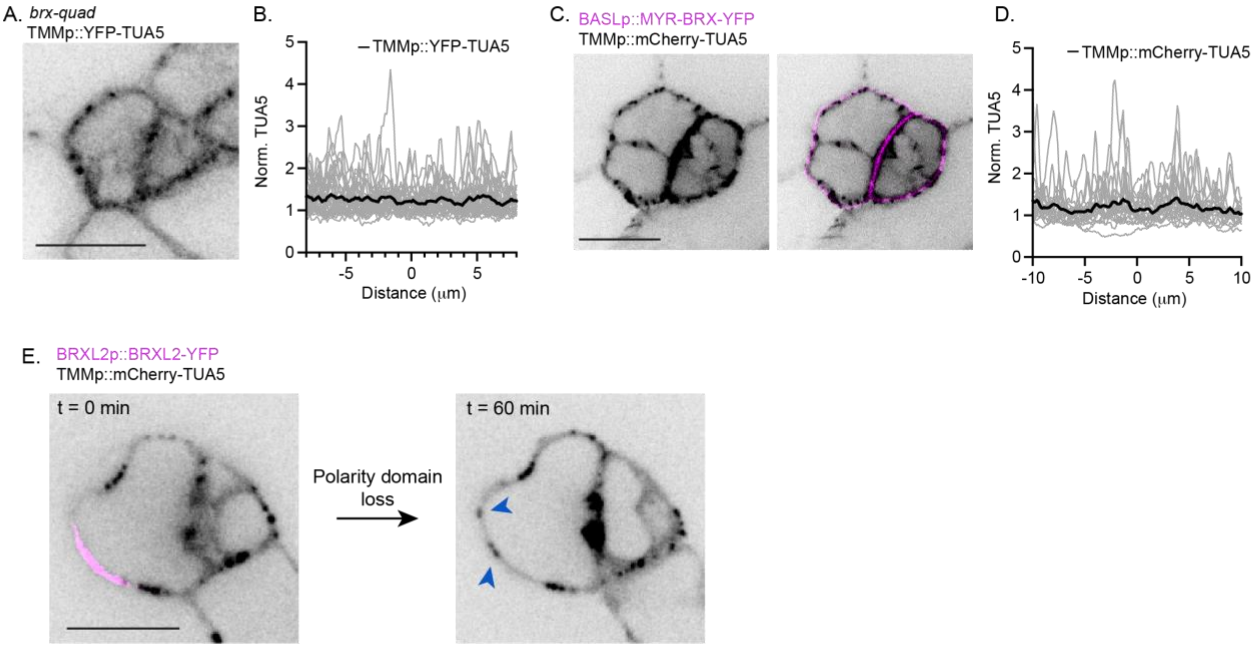

Fig. S7. Additional characterization of the interactions between stomatal lineage cell polarity domains and microtubule organization.

A. Representative medial section of an SLGC and associated meristemoid in a 3dpg *brx-quad* TMMp::YFP-TUA5 cotyledon. Scale bar-10µm.
B. Microtubule distribution in *brx-quad* SLGCs (gray lines) (n=31 cells). The black line shows the average microtubule signal.
C. Representative optical section at mid-cell height of an SLGC and associated meristemoid in a 3dpg BASLp::BRX-YFP TMMp::mCherry-TUA5 cotyledon. Scale bar-10µm.
D. Microtubule distribution in BASLp::MYR-BRX-YFP (n=22 cells). The black line shows the average microtubule signal.
E. Stills from a time-lapse movie showing cortical microtubule re-establishment (blue arrows) into a formerly polarized domain in an SLGC. Scale bar – 10µm.

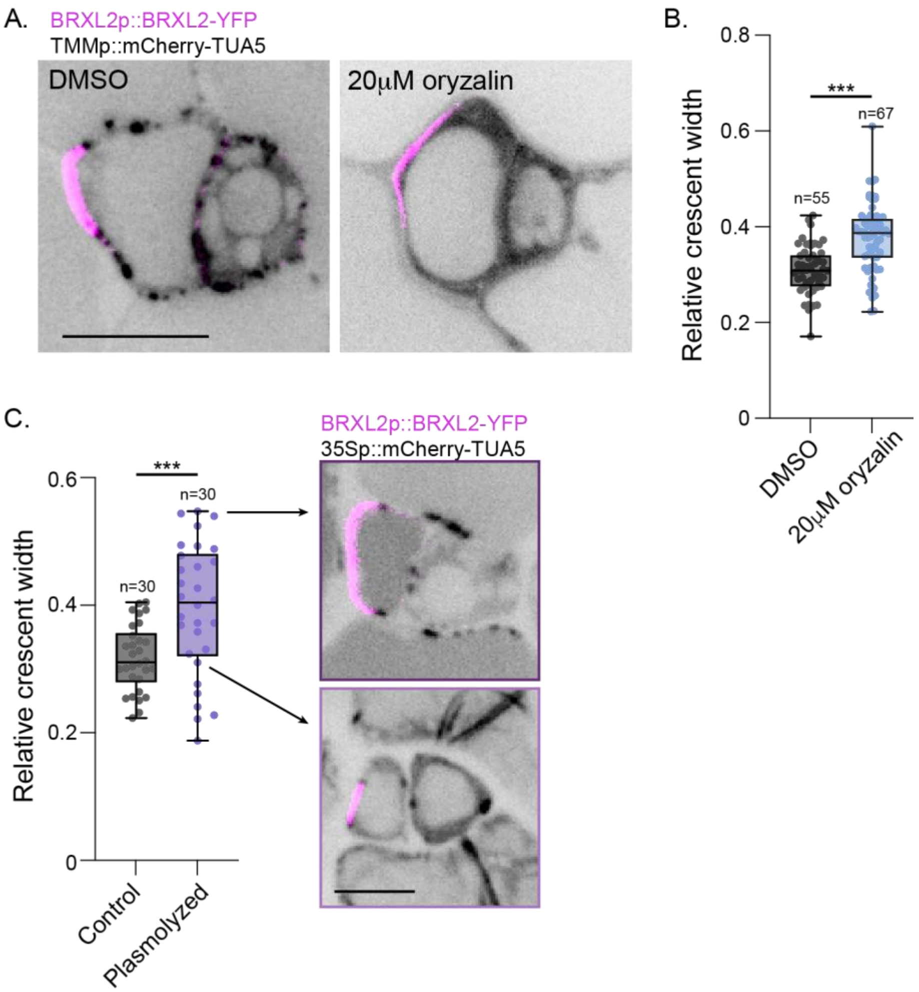

Fig. S8. Microtubule distribution sculpts polarity domain shape.

A. Representative images of a polarized SLGC and associated meristemoid in DMSO- and 20μM oryzalin-treated wild-type BRXL2p::BRXL2-YFP TMMp::mCherry-TUA5 seedlings. Scale bar-10μm.
B. Quantification of crescent widths in DMSO- (n=55) and 20μM oryzalin-treated (n=67) cells. Crescent widths were measured as a fraction of the total cell periphery. Unpaired t-test - $p<0.0001$.
C. Quantification of BRXL2 crescent widths in control or mildly plasmolyzed (n=30 cells each) BRXL2p::BRXL2-YFP 35Sp::mCherry-TUA5 cotyledons. Images on the right show examples where microtubules still mark the boundaries of the polarity domain despite accompanying changes in crescent width. Unpaired t-test - $p=0.0006$. Scale bar-5μm.

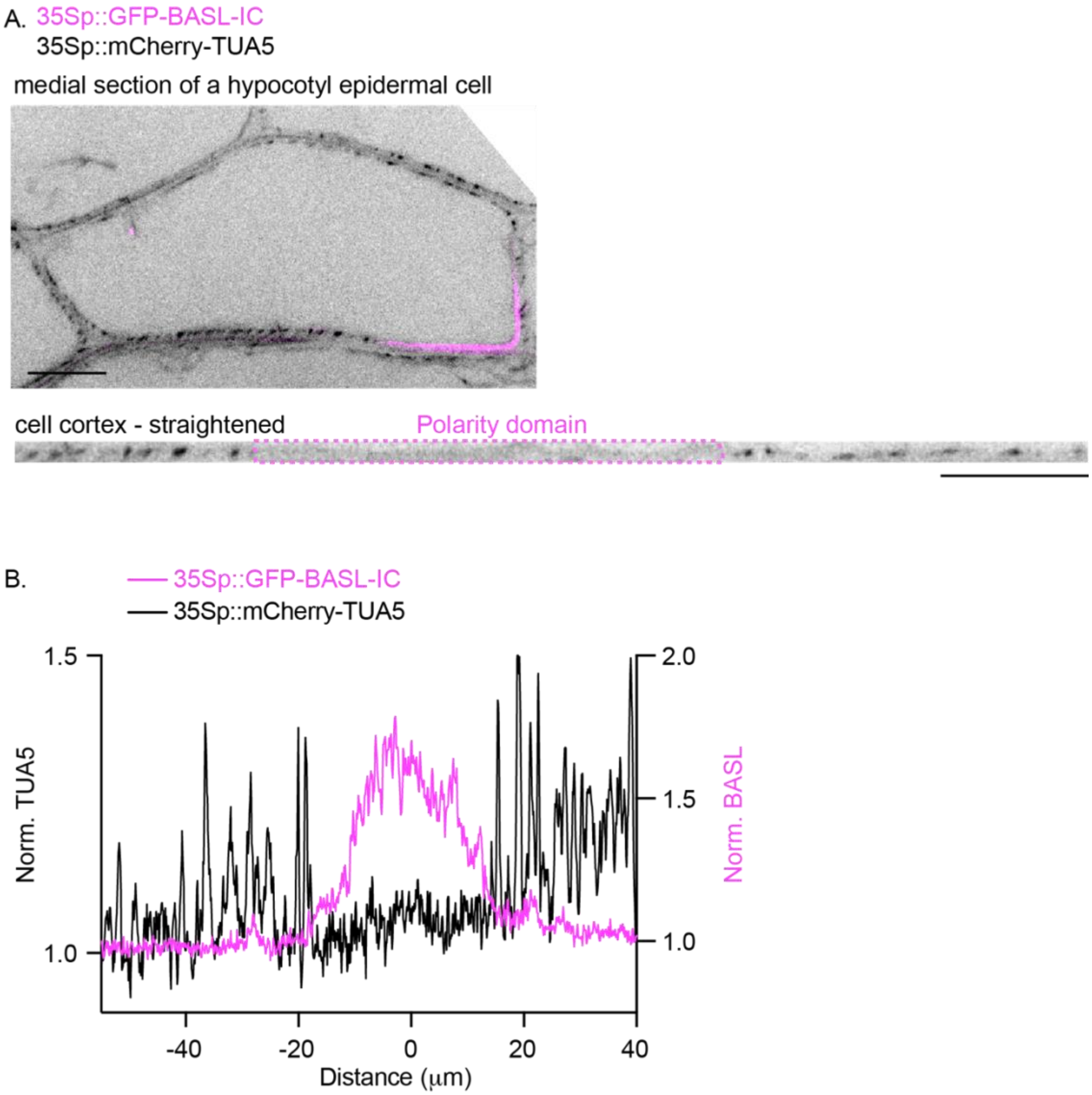

Fig. S9. BASL-IC overexpression locally depletes microtubules along the anticlinal wall in the hypocotyl epidermis.

A. (Top) Representative image of the medial section of a hypocotyl epidermal cell expressing 35Sp::GFP-BASL-IC and 35Sp::mCherry-TUA5. (Bottom) Straightened cell cortex showing the relationship between microtubule distribution and the ectopic BASL domain (outlined by the dotted magenta box). Image is representative of 44 examined cells. Scale bars-10μm.
B. Line scan along the cortex of the cell from (A), showing the fluorescence intensities of 35Sp::GFP-BASL-IC and 35Sp::mCherry-TUA5.

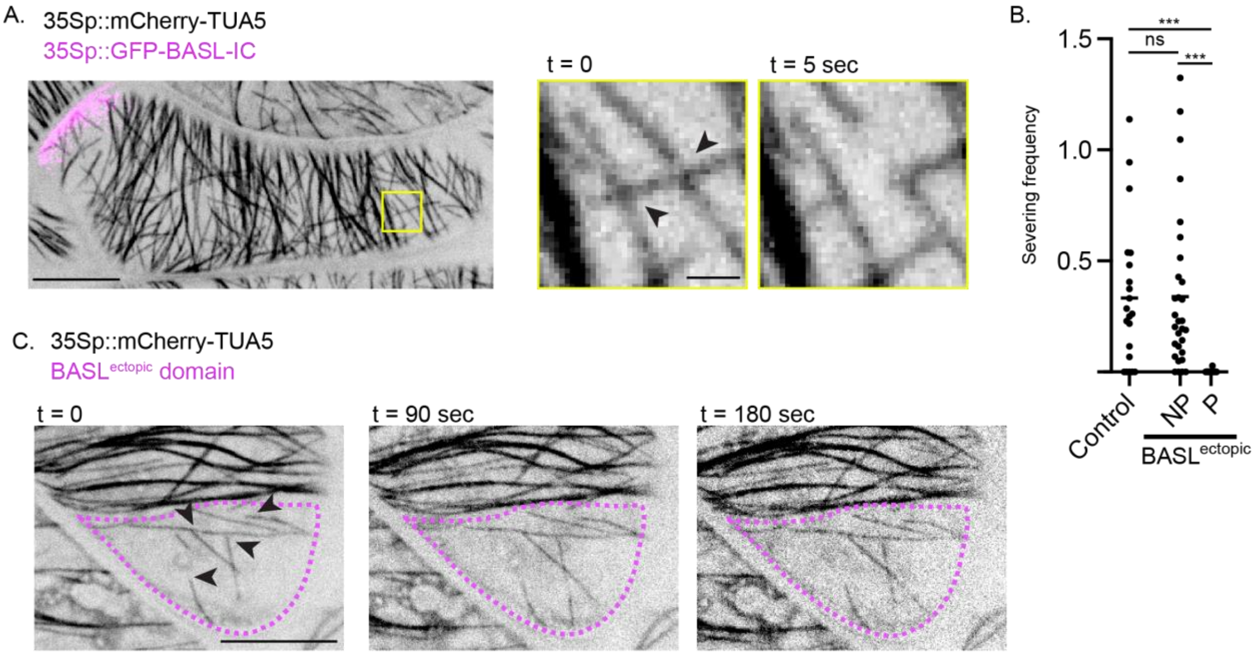

D.

	Polymerization rate (μm/min)	Depolymerization rate (μm/min)
Non-polarized region	3.594 ± 1.189 (n=111)	12.41 ± 5.364 (n=100)
Polarized region	2.841 ± 1.169 (n=114)	6.128 ± 3.072 (n=106)

Fig. S10. The BASL polarity domain does not locally deplete cortical microtubules by increasing microtubule severing or destabilizing microtubule minus ends.

A. Images showing an example of two severing events (arrows) at microtubule cross-over sites within the non-polarized area of a polarized hypocotyl epidermal cell. Scale bar (left)-10μm. Scale bar (right)-1μm.

B. Severing frequencies (# of severing events/100μm^2/min) from 30 BASL^{ectopic} (35Sp::GFP-BASL-IC 35Sp::mCherry-TUA5) cells (non-polarized (NP) and polarized (P) regions from the same cell) and 21 control (35Sp::mCherry-TUA5) cells. As severing events occur predominantly at microtubule cross-over sites and there are few cross-overs in polarized regions, severing frequencies are very low within BASL^{ectopic} domains. One-way ANOVA and Tukey's post hoc test – n.s. – not significant, *** - p = 0.0001.

C. Frames from a three-minute movie showing the stability of microtubule minus ends (arrows) within the ectopic polarity domain (dotted magenta line). Scale bar-10μm.

D. Microtubule plus-end dynamics in non-polarized and polarized regions in 35Sp::GFP-BASL-IC 35Sp::mCherry-TUA5 hypocotyl epidermal cells. Related to Fig. 4, D to F. Data are shown as the mean ± standard deviation.

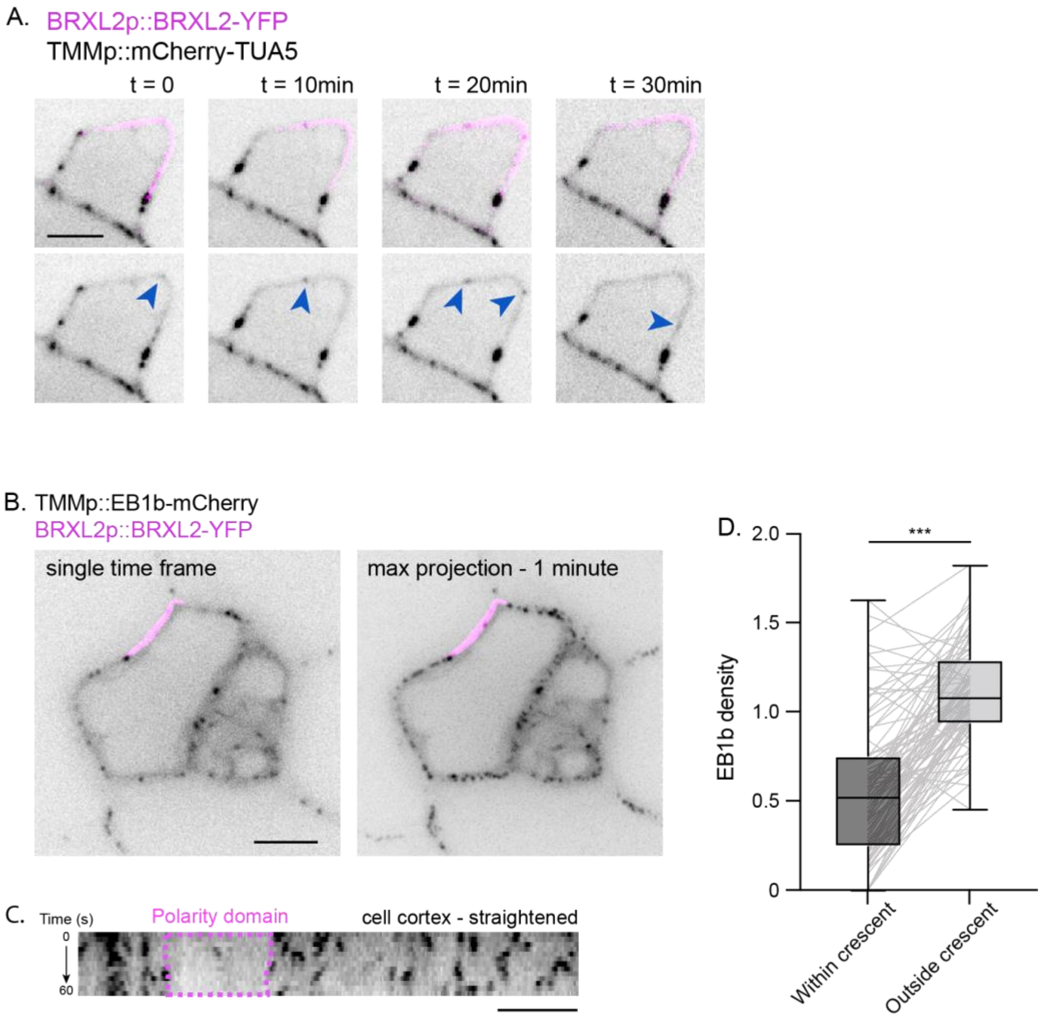

Fig. S11. Microtubules within the polarity domain are transient.

A. Stills from a time-lapse movie showing transient microtubules within the BRXL2 domain in a BRXL2p::BRXL2-YFP TMMp::mCherry-TUA5 cotyledon. The blue arrows indicate transient microtubules that appear in single time frames. Scale bar-5µm.
B. (Left) Single time point from a time-lapse move of an SLGC and associated meristemoid in a 3dpg BRXL2p::BRXL2-YFP TMMp::EB1b-mCherry cotyledon. (Right) Maximum projection of a one-minute movie of EB1b dynamics (tracking polymerizing microtubule plus ends). Scale bar-10µm.
C. Kymograph of the straightened cell cortex of the cell in (A) showing EB1b dynamics at medial height over one minute. The BRXL2 polarity domain is represented by the dotted magenta box. Scale bar-10µm.
D. Quantification of EB1b density (# of EB1b puncta/10µm/min) within and outside the BRXL2 domain (n=87 cells). For each of the cells, the EB1b density within and outside the crescent were quantified. Grey lines connect the two measurements made for a given cell. Overlaid box and whisker plots show the population values for the mean, 25th and 75th percentiles, and minimum and maximum values. In 78/87 cells, the EB1b density was higher outside the crescent than within it. Paired t-test – $p < 0.0001$.

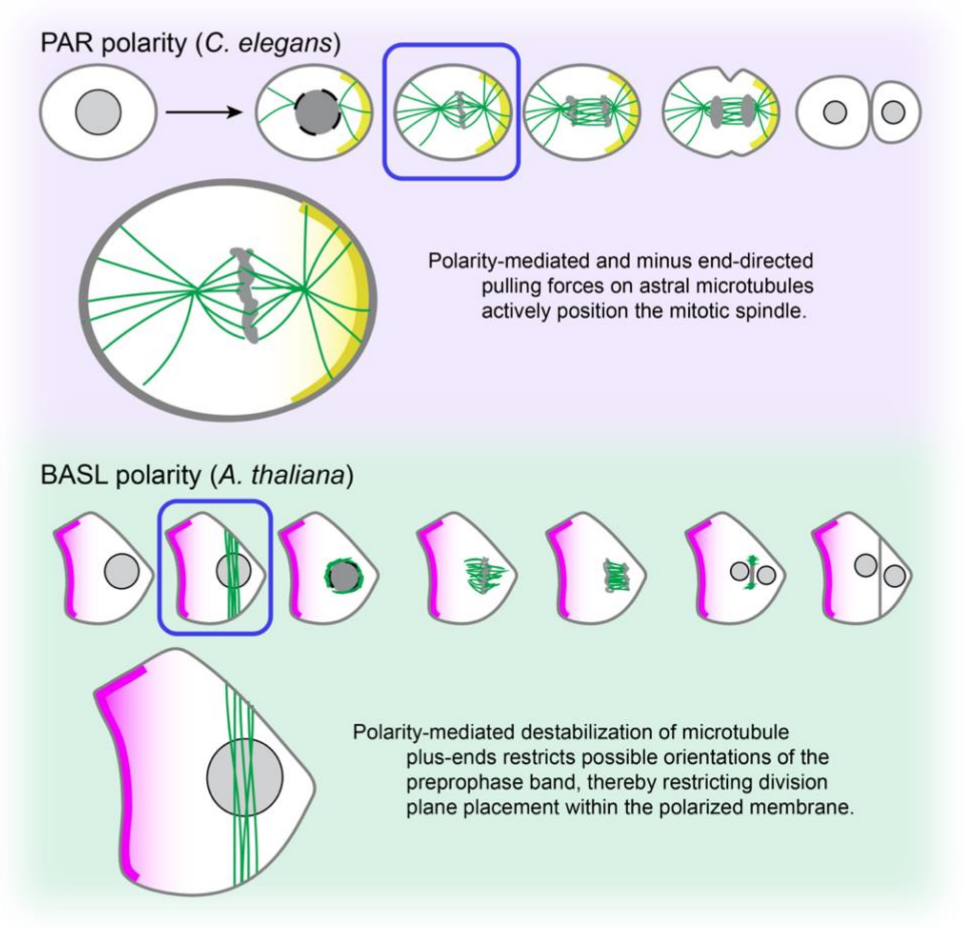

Fig. S12. Comparison of polarity-mediated asymmetric fate segregation in animals and plants.
In animal cells, asymmetric partitioning of fate determinants through asymmetric cell division relies on coupling the localization of these fate factors to spindle orientation. In the canonical model (top), fate determinants are localized to one cell pole, and polarity-mediated pulling forces on astral microtubules control spindle position and downstream division orientation. Because this pathway relies on length-dependent interactions between spindle-derived microtubules and the cortex, cells utilizing this spindle-positioning mechanism must be morphologically homogenous to yield predictable and robust division outcomes.

By contrast, asymmetrically dividing cells in the *Arabidopsis* stomatal lineage are morphologically varied but, nonetheless, must ensure asymmetric inheritance of the BASL polar crescent. In our model, the polar domain induces local depolymerization of cortical microtubules to control preprophase placement and division orientation. Importantly, this model can accommodate morphologically heterogenous cells because it does not require that the cell use the polarized site to directly specify the division site (as in the animal cell model). Instead, the polar domain is used to control where the division plane *cannot* be placed while allowing the default division pathways to control division orientation within this constraint. Therefore, animal and plant cells both harness polarity-microtubule interactions to control singular inheritance of fate regulators but utilize opposing logic.
